# A Preference for Peers over Faculty in the Pandemic Era: Development and Evaluation of a Medical Student-led Virtual Physiology Exam Review

**DOI:** 10.1007/s40670-021-01478-6

**Published:** 2021-12-02

**Authors:** Arina Alexeeva, Abigail R. Archibald, Joseph A. Breuer, Milton L. Greenberg

**Affiliations:** 1 School of Medicine, University of California, Irvine, CA 92697 USA; 2 Institute for Clinical and Translational Science, University of California, Irvine, CA 92697 USA; 3 Department of Physiology and Biophysics, Medical Science D350, University of California, Irvine, CA 92697 USA

**Keywords:** Peer-assisted learning, Undergraduate medical education, Remote learning, COVID-19, Physiology education

## Abstract

In the middle of the COVID-19 pandemic, students at the University of California, Irvine, reimagined their peer-led, small-group, tutorial sessions into an online format. The virtual sessions improved student-reported understanding of physiological principles and reduced exam anxiety. Peer-led review remains a valuable resource in the era of virtual medical education.

The COVID-19 pandemic disrupted many aspects of pre-clerkship medical education at the University of California Irvine, School of Medicine (UCISOM), including peer-led exam review sessions, a form of peer-assisted learning (PAL), for first-year medical students (MS1s). PAL has broad benefits in undergraduate medical curricula [1], and MS1s prefer peer-led review sessions over faculty-led sessions, reporting improved understanding and reduced exam anxiety when learning from their second-year peers [2]. With the abrupt COVID-19 school closures, UCISOM, like many universities, rapidly transitioned to fully remote teaching for pre-clerkship courses [3]. This transition to remote teaching included converting UCISOM’s peer-led physiology exam review sessions to a dynamic online version utilizing the Live-Zoom™ breakout room modality. In the current study, we evaluated whether the previously demonstrated preference for PAL pre-exam review sessions remained valid in this era of virtual medical education.

In the fall of 2020, MS1s at UCISOM attended virtual, peer-led and faculty-led review sessions to prepare for upcoming physiology block examinations. Peer-led sessions were taught by six MS2 tutors via a rotating Zoom™ breakout room format. MS1 students were divided into six breakout rooms while MS2 peer tutors rotated between rooms and reviewed faculty-approved exam-relevant physiology topics. Tutors spent 20 min in each breakout room and devoted a third of that time to answering MS1 questions. Faculty-led review sessions consisted of content review and practice questions in a large-group Zoom™ format.

To explore whether the previously demonstrated benefits of PAL persist when transformed into a digital platform, we surveyed MS1 perceptions of the online peer-led review session compared to the faculty-led sessions. The survey was modified from the published PAL questionnaire [2], consisting of eight items on a 4-point Likert scale. Student participation in the survey was optional, and 49% of the MS1 class submitted survey responses. We found that MS1s exhibited a strong preference for peer-led exam review sessions when compared to faculty-led sessions, despite the transition to a virtual format. The majority of MS1s reported that the peer-led sessions better helped them identify strengths and weaknesses, apply physiology concepts, reduce exam anxiety, and improve exam scores (Fig. 1). MS1s perceived the peer-presented content as more representative of exam questions than the content presented at faculty review sessions. Finally, the rotating breakout room format used in the peer-led sessions allowed students to better maintain focus and receive feedback. The ability to ask questions, however, was perceived as equivalent between the peer and faculty-led formats.

**Fig. 1:**
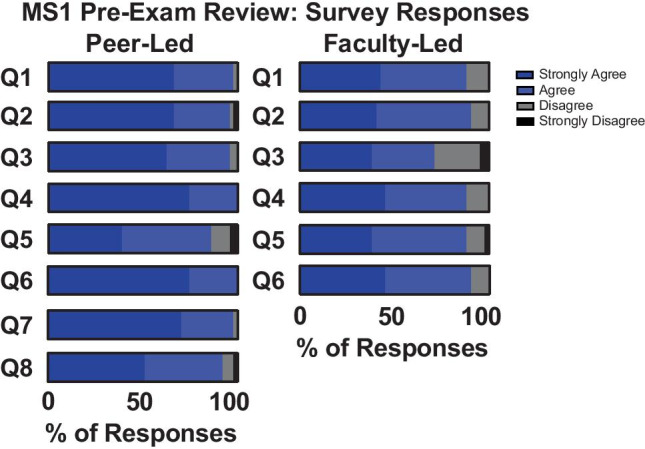
MS1 students’ survey responses. The remote, peer-led OR faculty-led tutorial session: (Q1) helped me identify my strengths and weaknesses in my understanding of medical physiology; (Q2) improved my understanding of and ability to apply physiological concepts; (Q3) reduced my anxiety regarding the upcoming exam; (Q4) improved my score on the physiology exam; (Q5) provided me with adequate opportunity to ask questions about course material and receive helpful feedback. (Q6) The content provided during the remote, peer-led OR faculty-led tutorial session was representative of the material on the exam. (Q7) Having several different peer tutors during the peer-led session facilitated my ability to remain focused and involved in the review session. (Q8) The smaller group size of the Zoom breakout room format (used in the peer-led tutorial session) facilitated my ability to ask questions and receive feedback. The survey was sent to the MS1 class following the physiology block exam, and responses to survey questions were anonymous, shared in aggregate form, with privacy and confidentiality maintained. This study was qualified as exempt research by the UCI Institutional Review Board for Human Subjects. The data includes 51 total survey responses from MS1 review session attendees, representing 49% of the entire MS1 class. Forty-two of these responses were from MS1s who attended both the peer-led and faculty-led review sessions, and the 9 remaining responses were from MS1s that only attended the peer-led review

Overall, our findings suggest that peer-led review sessions remain an acceptable alternative to faculty-led review sessions in the era of virtual learning, with students preferring peer-led review in various categories. This is consistent with the theory of cognitive congruence as it applies to PAL, which speculates that narrowing the knowledge gap between student and educator enhances learning. Factors such as usage of similar language between educators and students, as well as educators’ understanding of which concepts are important or difficult for learners to grasp, contribute to better educational outcomes [1, 4]. Our results reflect the advantages of PAL, demonstrating that student preference for peer-led sessions over faculty-led sessions did not change with the transition to a virtual platform.

In our previous study, we had inferred that student preference for peer-led review may in part be due to the utilization of a small-group format as it facilitates more direct interaction with the tutors [2]. Interestingly, despite continuing the small-group format in this study, MS1 students indicate that both virtual peer-led and faculty-led sessions provide a comparable degree of opportunity to ask questions and engage. This outcome may partially be due to usage of the private chat function on Zoom™, which eliminates a sense of disruption when asking questions, while also providing a level of anonymity. This is consistent with studies demonstrating that the pre-clerkship remote learning environment increases student engagement [5]. Considering this, the similar level of student engagement in both peer-led and faculty-led reviews seen here suggests that this transition to virtual review may have partially bridged the gap in student engagement that was observed during in-person faculty-led sessions [2]. Our findings demonstrate that small-group, peer-led medical physiology review sessions can be successfully translated onto virtual platforms and may continue to serve pre-clerkship learners into the future.

## Declarations

Student responses to survey questions were anonymous, shared in aggregate form, with privacy and confidentiality maintained. This study was qualified as exempt research by the UCI Institutional Review Board for Human Subjects.
